# Montelukast for Medical Delay in Flap Surgery

**DOI:** 10.29252/wjps.9.1.48

**Published:** 2020-01

**Authors:** Maryam Iranpour, Ali Khodarahmi, Nima Khodarahmi, Mohammad Shafiee, Reza Malekpourafshar, Nozar Nakhaee

**Affiliations:** 1 Department of Pathology, Pathology and Stem Cell Research Center, Kerman University of Medical Sciences, Kerman, Iran;; 2 Department of Surgery, Pathology and Stem Cell Research Center, Kerman University of Medical Sciences, Kerman, Iran;; 3 Physiology Research Center, Kerman University of Medical Sciences, Kerman, Iran;; 4 Department of Surgery HPB and Transplantation, Afzalipoor Hospital , Kerman, Iran;; 5 Neuroscience Research Center, Kerman University of Medical Sciences, Kerman, Iran

**Keywords:** Flaps, Medical delay, Montelukast

## Abstract

**BACKGROUND:**

Delay phenomenon can be used for better blood supply of the flap in plastic surgery. Effects of Montelukast have been observed to reduce ischemia/reperfusion injury in various organs due to angiogenic and anti-oxidant effects. The present study aimed to determine the role of Montelukast as medical delay of the flaps.

**METHODS:**

In this experimental study, 42 Wistar rats were divided into 3 equal groups. These groups were Surgical Delay Group (SDG), Medical Delay Group (MDG) and Control Group (CG). In SDG, 8×3 cm rectangular randomized random skin flap was first surgically delayed at rats’ back. The MDG received 10 mg/kg oral Montelukast via orogastric tube for 5 days as medical delay. In MDG and SDG flap, harvesting was undertaken after a delayed period, but there was not any delayed period in CG. After delayed period, a segment of the skin flap was biopsied for assessing angiogenesis. After 14^th^ days, the photos were taken and the size of the necrotic area of the flap was measured.

**RESULTS:**

A significant difference was observed between the mean survival and angiogenesis (*p*=0.002). The same performance was reported between MDG and SDG, which were alike regarding survival and angiogenesis (*p*>0.05); while there was a significant difference between the control and surgical groups, as well as control and medical groups (*p*<0.05). Finally, the inflammation showed no significant difference (*p*>0.05).

**CONCLUSION:**

Regarding positive effects of Montelukast on survival and angiogenesis, it is recommended to be used as a medication for larger studies.

## INTRODUCTION

Flap utilization has attracted a lot of attention in plastic surgery. Flaps are regarded as a complex of tissues with vascular supply. Random cutaneous flap is a skin flap harvested on the base of subdermal vascular plexus without innominated vessels. Lack of innominated vessels in random flaps leads to a higher necrosis rate than other flaps.^1^ Blood supply preservation is crucial to conserve the flap due to the significance of pedicle anatomy. However, blood conservation is considered as an important limitation, especially in random flaps. Each flap lost leads to an increased rate of operations, donor and receiver site deformity, limb loss risk, hospital stay, work withdrawal time, and disability.^2^

Surgical delay phenomenon which was first introduced in 1921 may be used for better blood supply. This phenomenon was used for flaps during 16^th^ century. Delay can result in circulating the flap better and increasing the survival by implementing surgical and medical methods. However, the efficacy of surgical method has been confirmed although the use of medical method is controversial. Two techniques are available for surgical method including standard which is done with or without pedicle preservation and strategic, which is performed with ligation of minor pedicles and transition after two weeks.^3^

The related mechanisms for surgical delay effect include an increase in flap tolerance to ischemia, the reconstruction of vascular tissue and an increase in flap blood supply, a decrease in arteriovenous shunt, vasoconstriction, and prothrombotic substance, and accordingly an increase in vascular extension of flap via choke arteries of flap and stimulation of angiogenesis by VEGF and FGF. Regarding the disadvantages of this method, we can refer to an increase in surgical stages, possible incorrect incision of flap pedicle, and scar formation leading to disordered flap rotation. Accordingly, medical method may be theoretically regarded as the optimal alternative.^3^

Some methods are implemented for medical delay development including, anti-inflammatory agents such as leukocyte aggregation and adhesion inhibitors, angiogenetic agents such as VEGF, PDGF, FGF, anti-spasmodic agents such as alpha-adrenergic antagonists, catecholamine release inhibitors, beta-agonist, direct vasodilators, and calcium channel inhibitors.^4^ Anti-coagulants, thrombolytics, and anti-spasmodics could play a significant role in some free flaps although no efficacy was found in other flaps in some other studies.^5^

Angiogenetic agents are useful for medical delay although they are not regarded as accessible medications and include high costs. Montelukast as a leukotriene inhibitor is used for treating asthma and allergy. Montelukast inhibits leukotrienes, which act as angiogenesis inhibitors. Further, the related effects of Montelukast reducing ischemia/reperfusion injury in various organs have been reported in some other studies, which is related to angiogenetic properties and anti-oxidant effects.^5^ However, no study, to the best of our knowledge, has evaluated this role in medical delay. Therefore, this study was undertaken to evaluate the Montelukast for medical delay in flap surgery.

## MATERIALS AND METHODS

In this experimental study, 42 Wistar rats weighing from 350 to 450 gram were randomly divided into three equal groups. These groups were Surgical Delay Group (SDG), Medical Delay Group (MDG) and Control Group (CG). All surgical procedures in these groups were conducted under general anesthesia with intramuscular administration of *ketamine* (20 mg/kg) and *xylotene* (2 mg/kg). Before each surgery, the single intramuscular dose of ceftriaxone (50 mg/kg) was administrated, which was repeated as single daily dose for 7 days after all surgeries and each rat’s back hair was shaved by an electrical shaver. After that, the skin was scrubbed by povidone-iodine solution. 

The flap was designed as an 8×3 cm rectangular, cephalic-based random skin flap at the back of rat. During the study, the animals were kept in a field with enough light and time of darkness. The temperature was preserved to optimal and their food was prepared. Each rat was kept in a separate cage and was cleaned regularly as described before.^6^ Regarding surgical group at the first stage, the flap was designed at the back of the rats. The skin was incised in all borders of flap except its base. In addition, the distal third of the flap was elevated from the fascia and the incisions of full-thickness skin was done till fascia. 

**Fig. 1:**
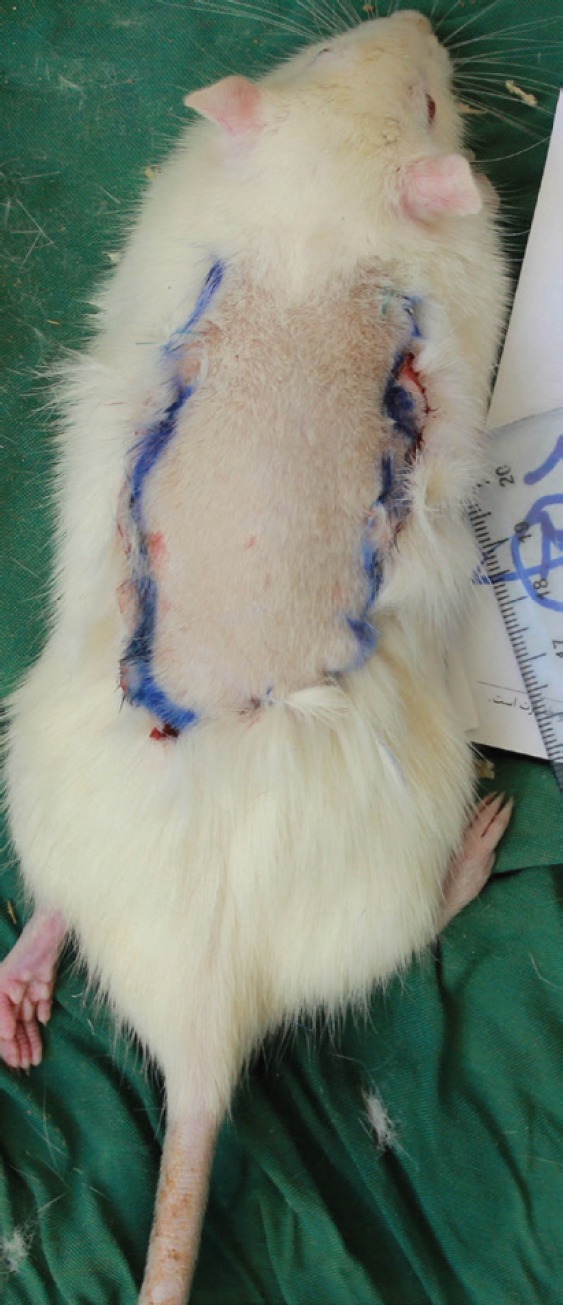
Practical procedures for surgical delay

At the end of this stage, hemostasis was conducted by a bipolar electrocautery and accordingly the skin flap was returned to its original site and sutured to the surrounding skin by 4/0 Nylon (Figure 1). During the second operation stage, all rats of this group underwent general anesthesia after 7 days. Then, all previous sutures of the flap were removed and a 3×3 mm segment of the most distal part of the viable flap was obtained with biopsy for assessing angiogenesis. The flap was completely elevated from the fascia; then it was returned to its original site after careful hemostasis and sutured to the surrounding skin by 4/0 nylon stitches. 

**Fig. 2:**
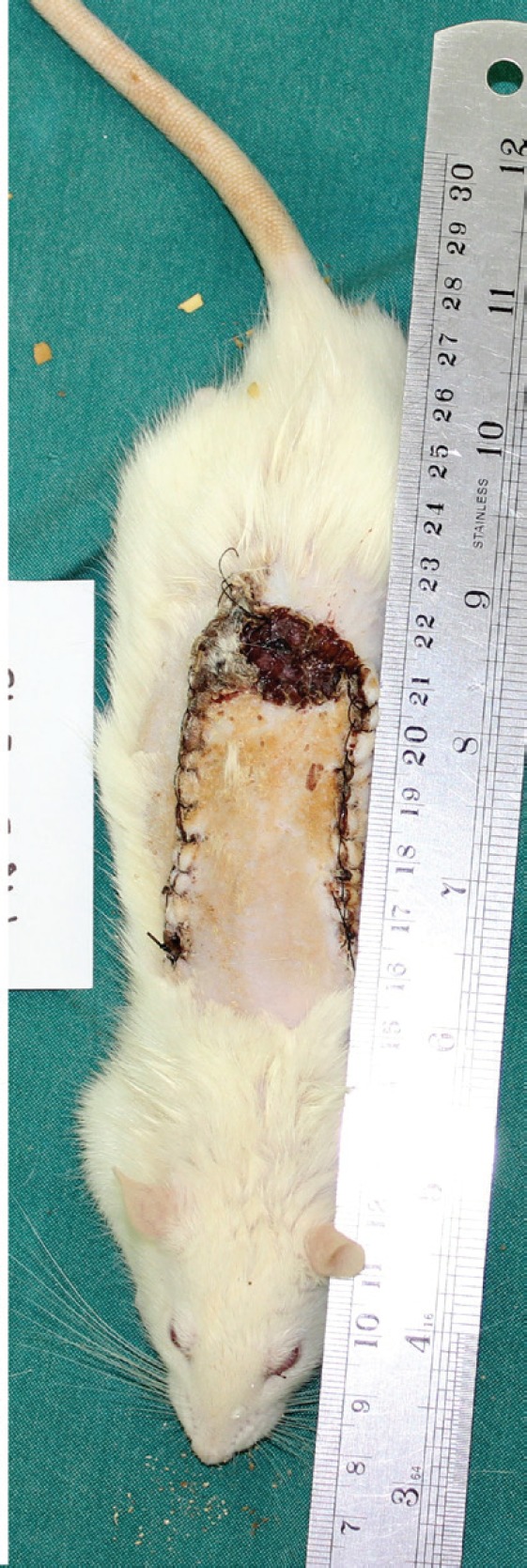
Final stage of flap study

After 14 days of the second stage operation, all rats of the SDG underwent general anesthesia. Furthermore, the photos of all flaps was taken by Nikon camera from 90 cm distance and a 3×3 mm biopsy was done from the most proximal part of the necrotic part of the flap for confirming necrosis (Figure 2). Regarding the medical group, the rats received Montelukast (10 mg/kg) as a single daily oral dose by gavage via orogastric tube for five days as medical dely. Then, all rats of MDG underwent general anesthesia during the first operation stage, 7 days after medical delay. 

The randomized skin flap was elevated from the fascia. Further, a 3×3 mm segment of the most distal part of the flap biopsy was obtained for assessing angiogenesis. After careful hemostasis, the flap was returned to its original site and sutured to the surrounding skin by 4/0 nylon stitches. Then, all rats of the medical group underwent general anesthesia after 14 days of the first stage operation. Accordingly, the photos of all flaps were taken by Nikon camera from 90 cm distance and a 3×3 mm incisional biopsy was done from the most proximal part of the necrotic part of the flap. This biopsy was used to confirm necrosis microscopically.

**Fig. 3:**
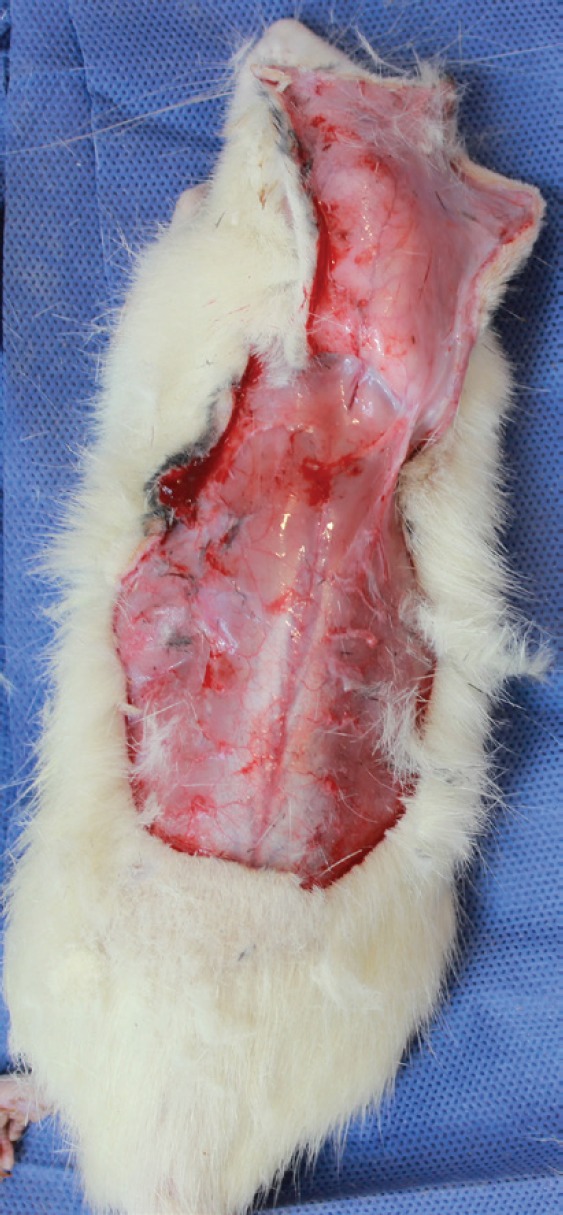
Practical procedure of flap harvesting

In the control group, all rats underwent general anesthesia without any delay procedure. The randomized skin flap was elevated from the fascia and a 3×3 mm segment of the most distal part of the flap was obtained for biopsy to assess angiogenesis. After careful hemostasis, the flap was returned to its original site and sutured to the surrounding skin by 4/0 nylon stitches (Figure 3). After 14 days of the original surgery, all rats of the CG underwent general anesthesia, photos was taken and a 3×3 mm segment of the proximal part of the necrotic area of the flap was biopsied for confirming necrosis.

Then samples for assessing angiogenesis were stained with H&E and CD31 staining. The angiogenesis was assessed by measuring new vessels under power field of 40 and samples for confirming necrosis were stained with H&E staining and necrosis was evaluated. The success rate of flaps was determined with flap photography, as well as measuring the remaining live flap surface with Image-J instrument. Based on the vessels per HPF, the angiogenesis was divided into mild (<5), moderate (6-10), and severe (>10) group in high power filed.^7^ In order to analyze the related data, Mann-Whitney, Kruskal-Wallis, Chi-Square, and Exact-Fisher tests were used and the significance level was reported to be 0.05.

## RESULTS

**Fig. 4:**
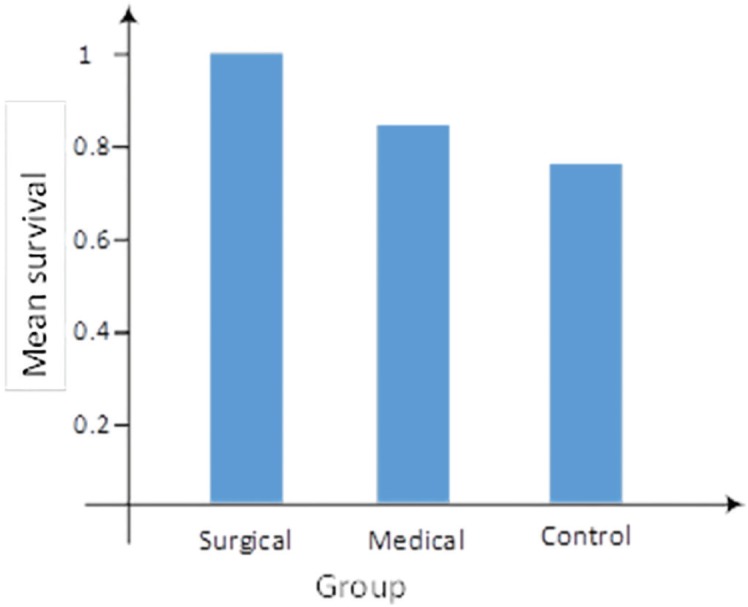
Mean survival rate across the groups

As shown in Figure 4, the mean survival was 0.95±0.11, 0.88±0.19, and 0.71±0.25 in surgical, medical, and control groups, respectively. In addition, the results of Kruskal-Wallis test indicated a significant difference (*p*=0.002). Further, the results of Mann-Whitney test showed that medical and surgical groups has the same level of survival (*p*=0.153). However, significant difference was observed between control and surgical groups (*p*=0.001), as well as between control and medical groups (*p*=0.022).

**Fig. 5:**
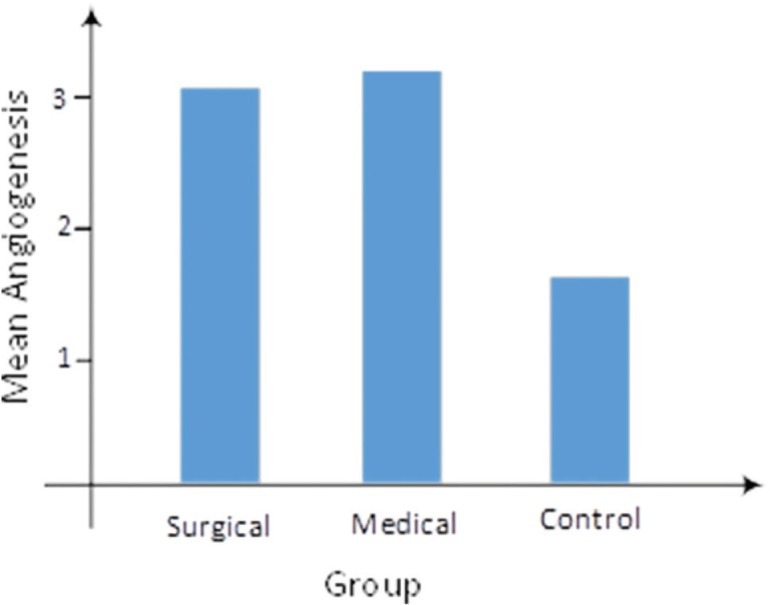
Mean angiogenesis rate across the groups

**Fig. 6:**
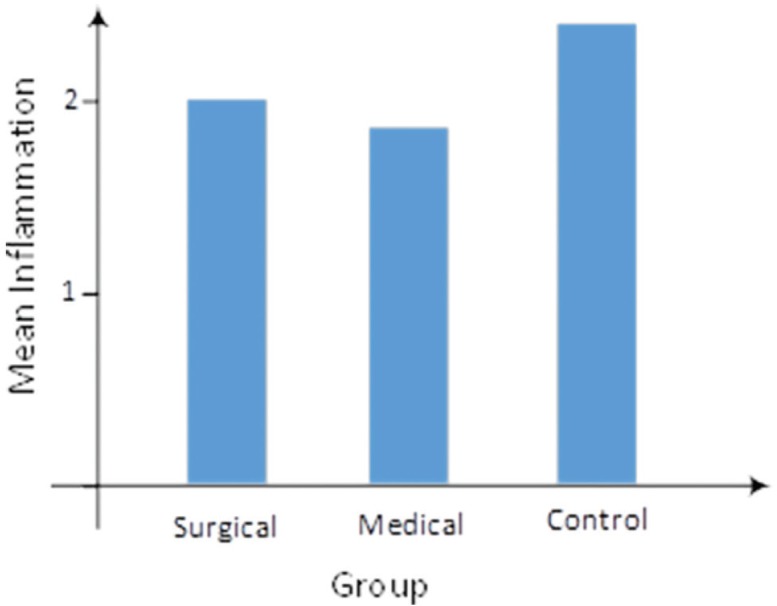
Mean inflammation rate across the groups

As illustrated in Figure 5, the mean angiogenesis was 3.08±0.76, 3.31±0.63, and 1.71±0.47 in surgical, medical, and control groups, respectively. In other words, a significant difference was observed according to Kruskal-Wallis test (*p*=0.0001). In addition, the Mann-Whitney test showed that medical and surgical groups had the same performance for angiogenesis (*p*=0.479). However, a significant difference was reported between control and surgical groups (*p*=0.0001), as well as between control and medical groups (*p*=0.0001). The mean inflammation was 2.08±0.95, 1.85±1.07, and 2.36±0.93 in surgical, medical, and control groups, respectively (Figure 6). In fact, no statistically significant difference was noted according to Kruskal-Wallis test (*p*=0.337).

## DISCUSSION

The present study aimed to evaluate the role of Montelukast as the medical delay of flaps. To this aim, the survival, angiogenesis, and inflammation of a randomized skin flap were compared between three equal groups based on surgical delay, medical delay, and control groups of Wistar rats. As it was already mentioned, surgical methods for delay flaps was a standard method of delay although may result in creating some adverse effects. In addition, finding good medical methods for delay may lead to better outcome for delay in flap applied surgeries. Based on the results, no significant difference was observed in the performance of survival, angiogenesis, and inflammation between surgical and Montelukast (medical) group. Accordingly, Montelukast may be used alternatively with good expected results. However, the inflammation was the same between all three groups, which was regarded as the least affected factor by Montelukast.

Celik *et al.* reported that oral Montelukast in rats prevented from ischemia and reperfusion injuries in healing process of wound in colon anastomosis after superior mesenteric artery obstruction.^8^ This issue is considered as a possible explanation for higher survival in Montelukast group, compared with control group. In the study of Lafci *et al.*, Iloprost and Montelukast therapies resulted in decreasing spinal injury after ischemia/reperfusion due to aortic clump, which may be evaluated in future studies regarding the possible drugs with synergistic effects; which may be used for medical flap delay.^9^

Wang *et al.* reported the protective role of Montelukast on neuronal ischemic injuries.^10^ In addition, Zhao *et al.* emphasized that Montelukast resulted in protecting rat brain against focal transient ischemia due to the obstruction of middle cerebral artery.^11^ Based on the results of these two studies, the possible central role of Montelukast was observed, along with the peripheral function of this drug which can attenuate the observed effects. In another study, Oral *et al.* reported that Montelukast lead to a decrease in the effect of ischemia/reperfusion injury in ovaries of the rats in congruent with the findings in the present study.^12^

In addition, Turtay *et al.* showed that Montelukast resulted in enhancing the rate of angiogenesis and faster wound healing process.^13^ Further, according to Ozturk *et al.*, Montelukast played the role of anti-oxidant leading to lower ischemia-reperfusion injury effects in testicular tissue in rats.^14^ Lower necrosis and higher survival can be regarded as indirect effects of this scavenging. In another study, Xu *et al.* demonstrated that CysLt receptor resulted in inhibiting angiogenesis. In addition, the inhibitors of this receptor such as Montelukast may lead to angiogenesis.^15^

Furthermore, Ozkan *et al.*^16^ indicated that Montelukast lead to lower ischemia/reperfusion injuries in hepatic tissue, which is in line with the results of flap delay process in the present study. Additionally, Daglar *et al.* emphasized a high potency of this medication for medical delay in flaps after the total obstruction of hepatic vessels in rats.^17^ In addition, Gideroglu *et al.* reported the lowered rate of ischemia/reperfusion injuries in axial skin flaps in rats by using montelukast, which is consistent with high survival in montelukast group in the present study.^18^

Yang *et al.*^19^ in another study demonstrated higher VEGF and VEGFR and better angiogenesis rate, which is congruent with the results of clinical animal in this study. Further, the results of the present study is in line with the study of Gideroglu *et al.*, which reported higher survival of random flap by using Montelukast at removing flap and up to six days.^20^ Finally, in another two studies by Sener and colleagues,^21^^,^^22^ good effects against ischemia-reperfusion injury in bladder and kidney tissues are in congruent with the findings for medical delay in flaps in the present study. In general, the results of the present study indicated good performance of Montelukast on survival and angiogenesis use of this medication for developing delay for skin flaps. However, further studies can be conducted in other animal models.
